# Supplementary material for: Factors Influencing the Prevalence of Resistance-Associated Substitutions in NS5A Protein in Treatment-Naive Patients with Chronic Hepatitis C
Source: Biomedicines. 2020 Apr 7;8(4):80. doi: 10.3390/biomedicines8040080 (PMC7235841; doi:10.3390/biomedicines8040080)
Supplement: Supplementary file 1 [file biomedicines-08-00080-s001.pdf]

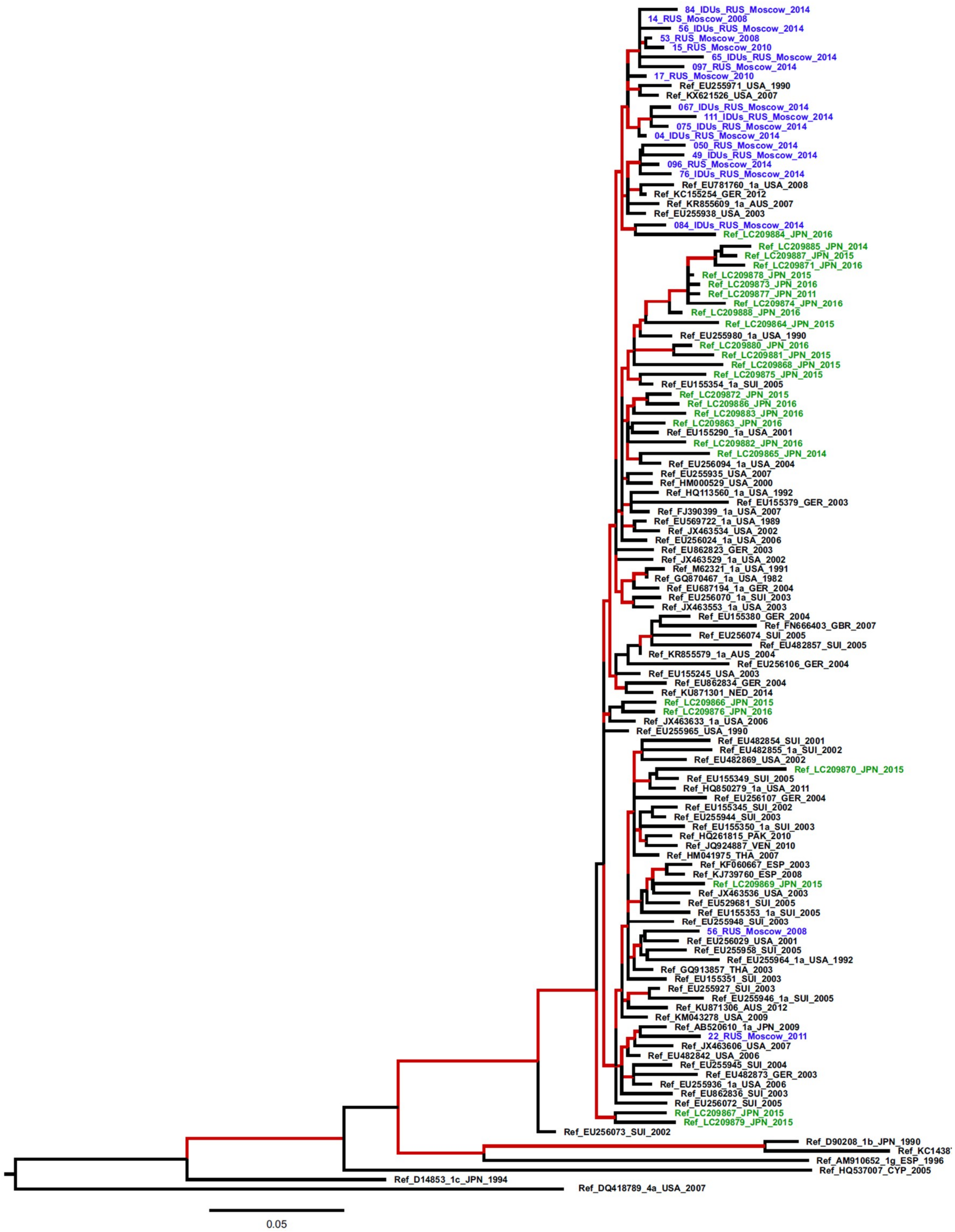

Supplementary Figure S1. Phylogenetic tree for HCV 1a core sequences (574 nucleotides, positions 342-915 according to HCV 1a reference strain H77 (Gene Bank accession number NC004102)), built under a GTR model; branches with group reliability >90% are indicated in red; HCV 1a sequences from this study are indicated in blue; HCV1a sequences from Japan [22] are indicated in green. Sequences from the intravenous drug users marked as IDUs.

a

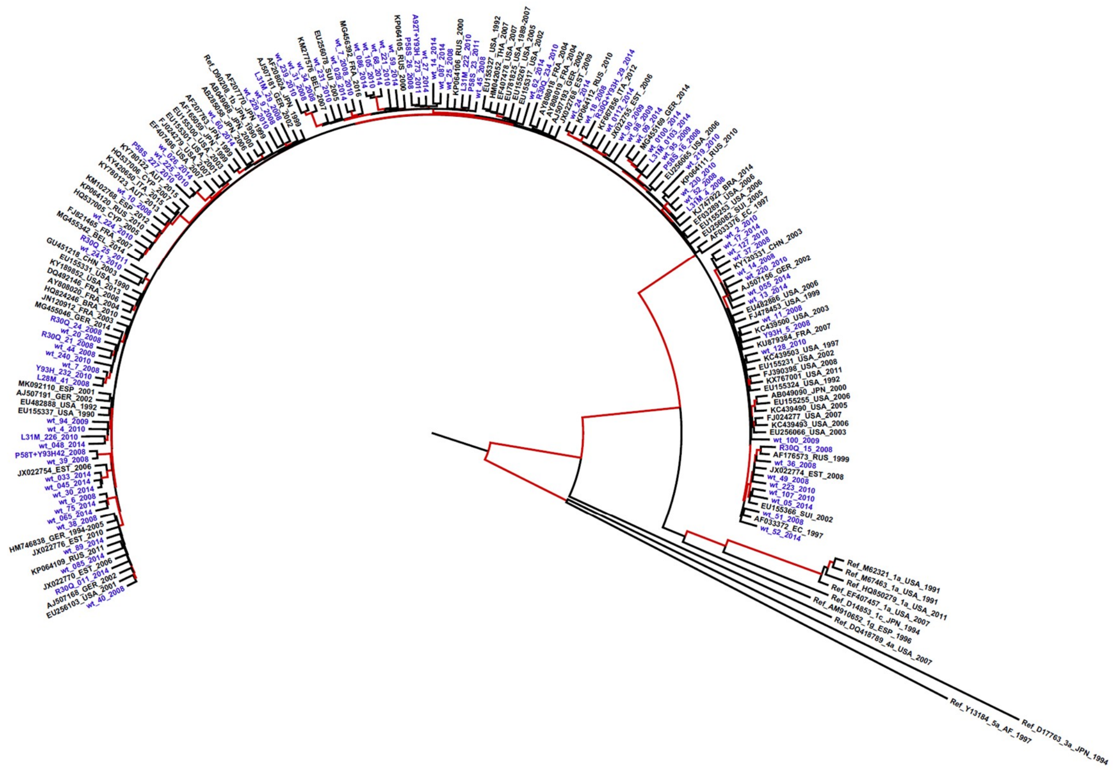

b

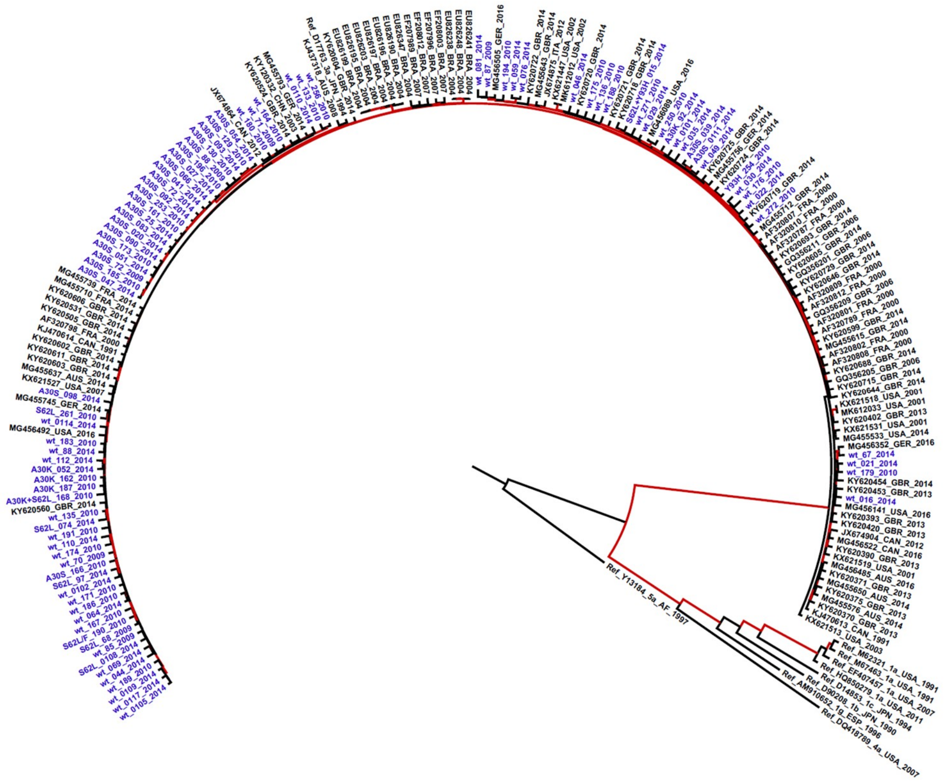

Supplementary Figure S2. Phylogenetic tree for HCV 1b (**a**) and HCV 3a (**b**) NS5A sequences (1126 nucleotides, positions 6330–7455 according to HCV 1a reference strain H77 (GenBank NC004102)), built under a GTR model; branches with group reliability >90% are indicated in red; sequences from this study are indicated in blue; the type of RAS or wild-type (WT) are indicated for each sequence.

Supplementary Table S1. Prevalence of resistance-associated amino acid substitutions in NS5A protein of HCV genotype 1a, 1b and 3a derived from the territory of the Russian Federation

**HCV genotype (nn of sequences)**	**Prevalence of NS5a resistance associated substitutions**			
	**Substitution**	**nn found**	**%**	**Resistance to***
1a (n=19)	**M28V**	11	57.9%	**OBV,** LDV, VEL
1b (n=93)	L28M	1	1.1%	EBR
	R30Q	7	7.5%	DCV, OBV, EBR
	**L31M**	5	5.4%	**DCV, LDV, EBR,** OBV, VEL
	P58S	4	4.4%	DCV, OBV
	P58T	1	1.1%	VEL
	A92T	1	1.1%	LDV, OBV
	**Y93H**	5	5.4%	**DCV, LDV, EBR,** OBV, VEL
3a (n=90)	**A30K**	5	5.7%	**PIB**,** DCV, VEL
	A30S	26	31.0%	DCV, VEL
	S62L	8	8.9%	DCV
	**Y93H**	2	2.2%	**PIB**,** DCV, VEL

DCV, daclatasvir; LDV, ledipasvir; EBR, elbasvir; OBV, ombitasvir; PIB, pibrentasvir; VEL, velpatasvir

Substitutions associated with > 10% of virologic failures are marked in bold.

*Resistance demonstrated both *in vitro* and *in vivo*.

** Dual variant A30K+Y93H results in significant resistance to PIB.

Supplementary Table S2. The list of HCV NS5A sequences from DAA treatment naïve patients retrieved from Gene Bank

#	Country of origin	Year	Gene bank accession number
**HCV-1a RAS M28V**			
1	USA/EU	before 2013	KC127126.1
2	USA/EU	before 2013	KC127209.1
3	USA/EU	before 2013	KC127211.1
4	USA/EU	before 2013	KC127219.1
5	USA/EU	before 2013	KC127232.1
6	USA/EU	before 2013	KC127235.1
7	USA/EU	before 2013	KC127280.1
8	USA/EU	before 2013	KC127305.1
9	USA/EU	before 2013	KC127394.1
10	USA/EU	before 2013	KC127568.1
11	USA/EU	before 2013	KC127573.1
12	USA/EU	before 2013	KC127592.1
13	USA/EU	before 2013	KC127608.1
14	Japan	2014	LC209876
15	Japan	2016	LC209874.1
16	Thailand	2007	HM042039
17	USA	2001	EU155285.2
18	USA	2001	EU155289.2
19	Germany	2003	EU155379.1
20	USA	2007	EU255935.1
21	USA	2003	EU255956.1
22	USA	1990	EU255982.1
23	USA	2005	EU256058.1
24	USA	2004	EU482847.1
25	Germany	2003	EU862823.1
26	Germany	2004	EU862824.1
27	USA	2008	FJ410172.1
28	USA	before 2008	EU781746.1
29	USA	before 2007	EF407456.1
30	Brazil or USA	before 2007	EF032884.1
**HCV-1a M28 (wild-type)**			
1	USA	1993	EU862827.1
2	USA	1989	EU862826.1
3	USA	1992	EU255992.1
4	USA	1989	EU255991.1
5	USA	1995	EU255988.1
6	USA	1989	EU255987.1

7	USA	1989	EU255986.1
8	USA	1991	EU255985.1
9	USA	1990	EU255983.1
10	USA	1990	EU255981.1
11	USA	1990	EU255980.1
12	USA	1991	EU255979.1
13	USA	1991	EU255978.1
14	USA	1991	EU255977.1
15	USA	1992	EU255976.1
16	Switzerland	2005	EU255930.1
17	Switzerland	2004	EU255929.1
18	Switzerland	2003	EU255928.1
19	Switzerland	2003	EU255927.1
20	Switzerland	2003	EU660385.1
21	Switzerland	2005	EU529681.1
22	USA	2004	EU529676.1
23	USA	1990	EU234065.2
24	Germany	2004	EU155380.2
25	Germany	2003	EU155378.2
26	Switzerland	2006	EU155355.2
27	Switzerland	2005	EU155354.2
28	Switzerland	2005	EU155353.2
29	Switzerland	2004	EU155352.2
30	Switzerland	2003	EU155351.2
31	Switzerland	2005	EU155349.2
32	Switzerland	2005	EU155348.2
33	Switzerland	2005	EU155346.2
34	Germany	2003	EU482873.1
35	Switzerland	2004	EU482872.1
36	USA	2002	EU482871.1
37	Switzerland	2001	EU482854.1
38	Switzerland	2005	EU482853.1
39	USA	1989	EU482852.1
40	USA	1990	EU482850.1
41	USA	2005	EU482848.1
42	Germany	2004	EU482832.1
43	Germany	2003	EU482831.1
44	Switzerland	2003	EU255959.1
45	Switzerland	2007	EU687195.1
46	Switzerland	2004	EU687194.1
47	Switzerland	2005	EU687193.1
48	Switzerland	2004	EU256107.1
49	Switzerland	2003	EU862836.1
50	Germany	2004	EU862834.1
**HCV-1b RAS Q30R**			
1	Japan	before 2007	AB285065.1

2	Japan	before2012	AB518783.1
3	Japan	before 2011	AB602003.1
4	Japan	before 1994	D63857.1
5	USA	2003	EU155228.2
6	USA	2006	EU155258.2
7	USA	2001	EU155262.2
8	Switzerland	2004	EU155361.2
9	Switzerland	2005	EU155374.2
10	USA	1992	EU234062.2
11	Switzerland	2003	EU256085.1
12	USA	2003	EU256088.1
13	USA	2006	EU256091.1
14	USA	2001	EU862835.1
15	USA	1996	HQ110091.1
16	China	before 2016	HQ639947
17	Germany	2008	HQ908333.1
18	Estonia	2007	JX022771.1
19	USA	2004	KC439485.1
20	Italy	2009	KX688410.1
21	Italy	2014	KX688434.1
22	USA	before 2007	EF407502
23	USA	before 2007	EF407472
24	USA	before 2007	EF407487
25	USA	before 2007	EF407501
26	Japan	before 2001	AB049100
27	Japan	before 2000	AF207756
28	USA/EU	before 2013	KC124806.1
29	USA/EU	before 2013	KC124813.1
30	USA/EU	before 2013	KC124839.1
31	USA/EU	before 2013	KC124845.1
32	USA/EU	before 2013	KC124886.1
33	USA/EU	before 2013	KC124903.1
34	USA/EU	before 2013	KC124927.1
35	USA/EU	before 2013	KC124940.1
36	USA/EU	before 2013	KC124945.1
37	USA/EU	before 2013	KC124951.1
38	USA/EU	before 2013	KC124960.1
39	USA/EU	before 2013	KC124978.1
40	USA/EU	before 2013	KC124983.1
**HCV-1b Q30 (wild-type)**			
1	USA/EU	before 2013	KC124769.1
2	USA/EU	before 2013	KC124770.1
3	USA/EU	before 2013	KC124772.1
4	USA/EU	before 2013	KC124776.1
5	USA/EU	before 2013	KC124778.1
6	USA/EU	before 2013	KC124779.1

7	USA/EU	before 2013	KC124781.1
8	USA/EU	before 2013	KC124786.1
9	USA/EU	before 2013	KC124787.1
10	USA/EU	before 2013	KC124789.1
11	USA/EU	before 2013	KC124790.1
12	USA/EU	before 2013	KC124791.1
13	USA/EU	before 2013	KC124792.1
14	USA/EU	before 2013	KC124794.1
15	USA/EU	before 2013	KC124796.1
16	USA/EU	before 2013	KC124798.1
17	USA/EU	before 2013	KC124799.1
18	USA/EU	before 2013	KC124800.1
19	USA/EU	before 2013	KC124802.1
20	USA/EU	before 2013	KC124804.1
21	USA	before 2006	EF407473.1
22	USA	before 2006	EF407474.1
23	USA	before 2006	EF407475.1
24	USA	before 2006	EF407476.1
25	USA	before 2006	EF407477.1
26	USA	before 2006	EF407478.1
27	USA	before 2006	EF407479.1
28	USA	before 2006	EF407480.1
29	USA	before 2006	EF407481.1
30	USA	before 2006	EF407482.1
31	USA	before 2006	EF407483.1
32	USA	before 2006	EF407484.1
33	USA	before 2006	EF407485.1
34	USA	before 2006	EF407486.1
35	USA	before 2006	EF407488.1
36	USA	before 2006	EF407489.1
37	USA	before 2006	EF407490.1
38	USA	before 2006	EF407491.1
39	USA	before 2006	EF407492.1
40	USA	before 2006	EF407493.1
41	USA	before 2006	EF407494.1
42	USA	before 2006	EF407495.1
43	USA	before 2006	EF407496.1
44	USA	before 2006	EF407497.1
45	USA	before 2006	EF407498.1
46	USA	before 2006	EF407499.1
47	USA	before 2006	EF407500.1
48	Switzerland	2006	EU255960.1
49	USA	1991	EU255961.1
50	USA	1991	EU255962.1
**HCV-3a RAS A30S**			

1	Australia	2007	KX621499.1
2	USA	before2013	JX674929.1
3	United Kingdom	2013	KY620378.1
4	United Kingdom	2013	KY620410.1
5	United Kingdom	2014	KY620528.1
6	United Kingdom	2014	KY620753.1
7	United Kingdom	2014	KY620757.1
8	United Kingdom	2014	KY620781.1
9	United Kingdom	2014	KY620851.1
10	United Kingdom	2014	KY620854.1
11	United Kingdom	2003	GQ356216.1
12	United Kingdom	2006	GQ356217.1
13	Pakistan	2012	KC415734.1
14	Pakistan	2012	KC415735.1
**HCV-3a A30 (wild-type)**			
1	United Kingdom	2006	GQ356200.1
2	United Kingdom	2006	GQ356201.1
3	United Kingdom	2006	GQ356202.1
4	United Kingdom	2006	GQ356203.1
5	United Kingdom	2005	GQ356204.1
6	United Kingdom	2006	GQ356205.1
7	United Kingdom	2006	GQ356207.1
8	United Kingdom	2006	GQ356208.1
9	United Kingdom	2006	GQ356209.1
10	United Kingdom	2006	GQ356210.1
11	United Kingdom	2006	GQ356211.1
12	United Kingdom	2007	GQ356212.1
13	United Kingdom	2006	GQ356213.1
14	United Kingdom	2006	GQ356214.1
15	United Kingdom	2006	GQ356215.1
16	United Kingdom	2003	GQ356216.1
17	United Kingdom	2006	GQ356217.1
18	Pakistan	2012	KC415734.1
19	Pakistan	2012	KC415735.1
20	Pakistan	2012	KC415736.1
21	Pakistan	2012	KC415737.1
22	Pakistan	2012	KC415739.1
23	Pakistan	2012	KC415740.1
24	Pakistan	2012	KC415743.1
25	Pakistan	2012	KC415748.1
26	Pakistan	2012	KC415753.1
27	Pakistan	2012	KC415754.1
28	Pakistan	2012	KC415756.1
29	France	before 2002	AF320787.1
30	France	before 2002	AF320788.1

31	France	before 2002	AF320789.1
32	France	before 2002	AF320791.1
33	France	before 2002	AF320792.1
34	France	before 2002	AF320793.1
35	France	before 2002	AF320794.1
36	France	before 2002	AF320795.1
37	France	before 2002	AF320796.1
38	France	before 2002	AF320797.1
39	France	before 2002	AF320798.1
40	France	before 2002	AF320799.1
41	France	before 2002	AF320800.1
42	France	before 2002	AF320801.1
43	France	before 2002	AF320802.1
44	France	before 2002	AF320803.1
45	France	before 2002	AF320804.1
46	France	before 2002	AF320805.1
47	France	before 2002	AF320806.1
48	France	before 2002	AF320807.1
49	France	before 2002	AF320809.1
50	France	before 2002	AF320811.1

Supplementary Table 3. Data on HCV NS5A T-cell epitopes retrieved from IEDB database.

№ aa position of NS5a	9	10	11	12	13	14	15	16	17	18	19	20	21	22	23	24	25	**26**	27	**28**	**29**	**30**	**31**	**32**	33	34	35	36	37	**38**	39	40	41	42	43	44	45	46	47	48	49	50	51	52	53	54	55	56	57	**58**	59	60	61	**62**	63	64	65	66	67	68	69	70	71	72	73	74	75	76	77	78	79	80	81	82	83	84	85	86	87	88	89	90	91	**92**	**93**	94	95	96	97	98	99	100	101	102	103	104	105	106	107	108	109	110
№ aa position of polyprotein of HCV	1981	1982	1983	1984	1985	1986	1987	1988	1989	1990	1991	1992	1993	1994	1995	1996	1997	**1998**	1999	**2000**	**2001**	**2002**	**2003**	**2004**	2005	2006	2007	2008	2009	**2010**	2011	2012	2013	2014	2015	2016	2017	2018	2019	2020	2021	2022	2023	2024	2025	2026	2027	2028	2029	**2030**	2031	2032	2033	**2034**	2035	2036	2037	2038	2039	2040	2041	2042	2043	2044	2045	2046	2047	2048	2049	2050	2051	2052	2053	2054	2055	2056	2057	2058	2059	2060	2061	2062	2063	**2064**	**2065**	2066	2067	2068	2069	2070	2071	2072	2073	2074	2075	2076	2077	2078	2079	2080	2081	2082
aa of H77 (AF009606)	W	D	W	I	C	E	V	L	S	D	F	K	T	W	L	K	A	*K*	L	*M*	*P*	*Q*	*L*	*P*	G	I	P	F	V	*S*	C	Q	R	G	Y	R	G	V	W	R	G	D	G	I	M	H	T	R	C	H	C	G	A	E	I	T	G	H	V	K	N	G	T	M	R	I	V	G	P	R	T	C	R	N	M	W	S	G	T	F	P	I	N	A	Y	T	T	G	P	C	T	P	L	P	A	P	N	Y	K	F	A	L

№	Epitope ID	Starting Position	Ending Position	Description	Antigen Name	Organism Name	Epitope Comments
1	593385	13	30	CTVLTDFKTWLQSKLLPR	NS5A protein, partial	Hepatitis C virus subtype 1b	no data*
2	593710	21	38	TWLQSKLLPRLPGVPFLS	NS5A protein, partial	Hepatitis C virus subtype 1b	no data*
3	593613	29	46	PRLPGVPFLSCQRGYKGV	NS5A protein, partial	Hepatitis C virus subtype 1b	no data*
4	593558	37	54	LSCQRGYKGVWRGDGIMQ	NS5A protein, partial	Hepatitis C virus subtype 1b	no data*
5	593485	45	62	GVWRGDGIMQTTCPCGAQ	NS5A protein, partial	Hepatitis C virus subtype 1b	no data*
6	593573	53	70	MQTTCPCGAQITGHVKNG	NS5A protein, partial	Hepatitis C virus subtype 1b	no data*
7	593366	61	78	AQITGHVKNGSMRIVGPR	NS5A protein, partial	Hepatitis C virus subtype 1b	no data*
8	593614	77	94	PRTCSNTWHGTFPINAYT	NS5A protein, partial	Hepatitis C virus subtype 1b	no data*
9	593491	85	102	HGTFPINAYTTGPCTPSP	NS5A protein, partial	Hepatitis C virus subtype 1b	no data*
10	593768	93	110	YTTGPCTPSPAPNYSRAL	NS5A protein, partial	Hepatitis C virus subtype 1b	no data*
11	72337	1981	2000	WDWICEVLSDFKTWLKAKLM	Genome polyprotein	Hepatitis C virus (isolate 1)	CTL
12	170047	1981	2000	WDWICTVLTDFKTWLQSKLL	polyprotein	Hepatitis C virus subtype 1b	no data*
13	6208	1985	1999	CEVLSDFKTWLKAKL	HCV-1	Hepatitis C virus subtype 1a	no data*
14	69752	1987	2006	VLSDFKTWLKAKLMPQLPGI	Genome polyprotein	Hepatitis C virus (isolate 1)	no data*
15	57191	1989	2003	SDFKTWLKAKLMPQL	HCV-1	Hepatitis C virus subtype 1a	no data*
16	8285	1990	1999	DFKTWLQSKL	Genome polyprotein	Hepacivirus C	CTL
17	146608	1991	1999	VLSDFKVWL	Genome polyprotein	Hepatitis C virus subtype 6a	CTL
18	16472	1991	2010	FKTWLKAKLMPQLPGIPFVS	HCV-1	Hepatitis C virus subtype 1a	no data*
19	168568	1991	2010	FKTWLQSKLLPRLPGVPFLS	polyprotein	Hepatitis C virus subtype 1b	CTL
20	108687	1993	2001	VVSDFKTWL	Genome polyprotein	Hepacivirus C	CTL
21	67273	1993	2007	TWLKAKLMPQLPGIP	HCV-1	Hepatitis C virus subtype 1a	no data*
22	69750	1993	2001	VLSDFKSWL	polyprotein	Hepatitis C virus genotype 3	CTL
23	2235	1997	2016	AKLMPQLPGIPFVSCQRGYK	Genome polyprotein	Hepatitis C virus (isolate 1)	no data*
24	2234	1997	2011	AKLMPQLPGIPFVSC	HCV-1	Hepatitis C virus subtype 1a	no data*
25	32030	1998	2006	KLMPQLPGI	polyprotein	Hepatitis C virus (isolate Taiwa	CTL
CTL	38556	2000	2008	LPKLPGVPF	Genome polyprotein	Hepacivirus C	CTL
27	49026	2001	2015	PQLPGIPFVSCQRGY	HCV-1	Hepatitis C virus subtype 1a	no data*
28	49027	2001	2020	PQLPGIPFVSCQRGYKGVWR	HCV-1	Hepatitis C virus subtype 1a	no data*
29	169521	2001	2020	PRLPGVPFLSCQRGYKGVWR	polyprotein	Hepatitis C virus subtype 1b	no data*
30	20393	2005	2019	GIPFVSCQRGYKGVW	HCV-1	Hepatitis C virus subtype 1a	no data*
31	70287	2006	2015	VPFFSCQRGY	Genome polyprotein	Hepacivirus C	CTL
32	70893	2009	2023	VSCQRGYKGVWRVDG	HCV-1	Hepatitis C virus subtype 1a	no data*
33	6896	2011	2030	CQRGYKGVWRVDGIMHTRCH	HCV-1	Hepatitis C virus subtype 1a	no data*
34	168271	2011	2030	CQRGYKGVWRGDGIMQTTCP	polyprotein	Hepatitis C virus subtype 1b	CTL
35	23212	2017	2031	GVWRVDGIMHTRCHC	HCV-1	Hepatitis C virus subtype 1a	no data*
36	67961	2021	2035	VDGIMHTRCHCGAEI	HCV-1	Hepatitis C virus subtype 1a	no data*
37	67962	2021	2040	VDGIMHTRCHCGAEITGHVK	HCV-1	Hepatitis C virus subtype 1a	no data*
38	168630	2021	2040	GDGIMQTTCPCGAQITGHVK	polyprotein	Hepatitis C virus subtype 1b	CTL
39	177697	2023	2040	GIMQTTCPCGAQIAGHVK	polyprotein	Hepatitis C virus subtype 1b	no data*
40	41680	2025	2039	MHTRCHCGAEITGHV	HCV-1	Hepatitis C virus subtype 1a	no data*
41	6377	2029	2043	CHCGAEITGHVKNGT	HCV-1	Hepatitis C virus subtype 1a	no data*
42	177727	2029	2046	GVMSTRCPCGASIAGHVK	polyprotein	Hepatitis C virus subtype 3a	no data*
43	6272	2031	2050	CGAEITGHVKNGTMRIVGPR	HCV-1	Hepatitis C virus subtype 1a	no data*
44	168258	2031	2050	CGAQITGHVKNGSMRIVGPR	polyprotein	Hepatitis C virus subtype 1b	CTL
45	1002	2033	2047	AEITGHVKNGTMRIV	HCV-1	Hepatitis C virus subtype 1a	no data*
46	63097	2051	2070	TCRNMWSGTFPINAYTTGPC	HCV-1	Hepatitis C virus subtype 1a	no data*
47	169807	2051	2070	TCSNTWHGTFPINAYTTGPC	polyprotein	Hepatitis C virus subtype 1b	CTL
48	32559	2053	2067	KNMWSGTFFINAYTT	Genome polyprotein	Hepatitis C virus (isolate H)	no data*
49	55032	2053	2067	RNMWSGTFPINAYTT	HCV-1	Hepatitis C virus subtype 1a	no data*
50	58254	2057	2071	SGTFPINAYTTGPCT	HCV-1	Hepatitis C virus subtype 1a	no data*
51	63643	2059	2073	TFFINAYTTGPCTPL	Genome polyprotein	Hepatitis C virus (isolate H)	no data*
52	47931	2061	2075	PINAYTTGPCTPLPA	HCV-1	Hepatitis C virus subtype 1a	no data*
53	47932	2061	2080	PINAYTTGPCTPLPAPNYTF	HCV-1	Hepatitis C virus subtype 1a	no data*
54	169490	2061	2080	PINAYTTGPCTPSPAPNYSR	polyprotein	Hepatitis C virus subtype 1b	CTL
55	76186	2065	2079	YTTGPCTPLPAPNYK	Genome polyprotein	Hepatitis C virus (isolate H)	no data*
56	76187	2065	2079	YTTGPCTPLPAPNYT	HCV-1	Hepatitis C virus subtype 1a	no data*
							* Not characterized in IEDB and original study

Aa positions associated with RAS are highlighted in blue. Aa sequences corresponding to epitopes are highlighted in pin
